# Multi-omics Mendelian randomization to identify novel immune therapeutic targets in benign prostatic hyperplasia

**DOI:** 10.1016/j.gendis.2025.101944

**Published:** 2025-11-19

**Authors:** Zhenpeng Zhu, Xing Ji, Wenyuan Leng, Weimin Hu, Chunru Xu, Xiaoyu Li, Jinqin Qian, Cuijian Zhang, Jian Lin

**Affiliations:** a Department of Urology, Peking University First Hospital, Beijing 100034, China; b Institute of Urology, Peking University, Beijing 100034, China; c National Urological Cancer Center, Beijing 100034, China; d Beijing Key Laboratory of Urogenital Diseases (Male) Molecular Diagnosis and Treatment Center, Beijing 100034, China

Benign prostatic hyperplasia (BPH) is the most common benign condition associated with lower urinary tract symptoms in aging males, significantly impacting their quality of life.^1^ Accumulating evidence suggests that BPH is intricately associated with chronic inflammation.^2^ With the availability of large-scale Genome-Wide Association Study (GWAS) data and advancements in analysis methods, we investigated the causal relationship between the immune-related genes and BPH using multi-omics quantitative trait loci data. Notably, butyrophilin subfamily 3 member A2 (BTN3A2) emerged as a priority level 1 candidate gene, demonstrating consistent correlations with BPH across all analytical levels. The identification of BTN3A2-associated mechanisms provides novel insights into tissue-specific immune modulation in BPH management.

In this study, the overall research workflow is illustrated in Figure 1A. GWAS data were primarily obtained from two databases, the UK Biobank and FinnGen, and were integrated multi-omics data for statistical Mendelian randomization (SMR) verification. A set of immune-related genes (IRGs) was sourced from the InnateDB and Immuport databases, comprising 2518 combined IRGs. Within the UK Biobank database, after removing single-nucleotide polymorphisms with a *P*-value greater than 0.05 for SMR and a heterogeneity in dependent instruments (HEIDI)-adjusted *P*-value (P-HEIDI) less than 0.01, a total of 205 IRGs with 517 CpG sites remained (Table S1). Then, after screening single-nucleotide polymorphisms with a false discovery rate (FDR) less than 0.05, we found a total of 19 IRGs with 42 CpG sites significantly associated with BPH (Fig. 1B). The relevant results were also verified in the FinnGen database (Table S2). In the subsequent analysis, a total of 7 IRGs with an FDR less than 0.05 and posterior probabilities of hypotheses 4 (PPH4) greater than 0.70 were deemed to be significant and exhibited a co-localization relationship, including BTN3A2, C4B, C4A, AGER, RNF5, APOM, and NEU1 (Fig. 1C; Tables S3 and S4). At the protein level, we discovered that BTN3A2 and BTN3A3 were significantly correlated with BPH. Nevertheless, PPH4 showed no significant colocalization analysis in the FinnGen database (Tables S5 and S6).

**Figure 1:**
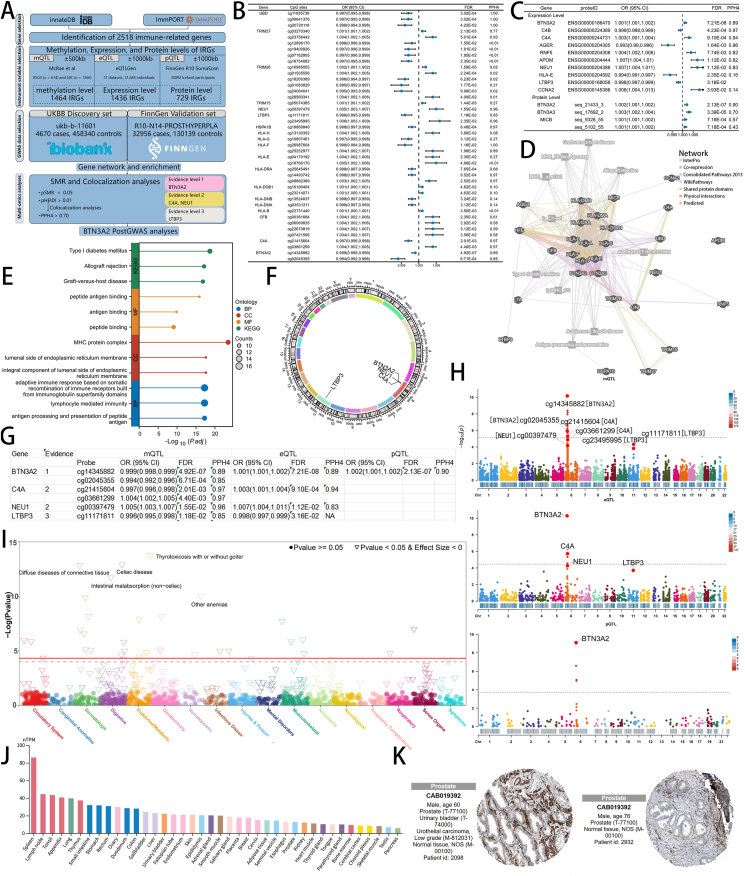
The findings of this study. **(A)** Flow chart of the overall analysis process. The overall experimental process includes the selection of gene(s) and instrumental variables. **(B)** The relationship between methylation levels of immune-related genes (IRGs) and benign prostatic hyperplasia (BPH) in the statistical Mendelian randomization (SMR) and colocalization analyses. **(C)** The relationship between expression and protein levels of IRGs and BPH in the SMR and colocalization analyses. **(D)** Interaction networks and potential related pathways, where different colors indicate different interaction types in the network. **(E)** GO and KEGG pathway enrichment analysis for significant immune-related genes, where circle size indicates how many genes are included and color indicates classification. **(F)** Chromosome circle diagram of evidence level genes with arrows pointing to gene chromosome locations. **(G)** The evidence level of IRGs with BPH at the methylation, expression, and protein levels. **(H)** The Manhattan plot between evidence level genes' features and BPH. **(I)** PheWAS analysis of BTN3A2 using the Lee_ukbb database, with different colors indicating different disease systems. **(J)** HPA database expression levels of BTN3A2 in different tissues, arranged from high to low. **(K)** The HPA database shows the expression level of the BTN3A2 protein in prostatic tissue, tissue information, and antibodies as indicated in the annotation.

Further, we selected 29 IRGs with SMR analysis FDR <0.05 in different omics levels for pathway enrichment analysis. We first analyzed the potential interaction network of these genes in GeneMania and identified their possible involved functions in the GeneMania database (Fig. 1D). Subsequently, Kyoto Encyclopedia of Genes and Genomes (KEGG) and Gene Ontology (GO) analyses were conducted on these IRGs, and the top 3 enriched pathways in each category were displayed. As was the case with the pathways enriched in GeneMania, these IRGs were most significantly enriched in the MHC complex pathway and may affect the binding of antigen to antibody based on the results of the cellular component outcomes (Fig. 1E; Table S7).

We categorized the causal effects of IRGs on BPH into three evidence levels based on the results: i) Genes with evidence level 1 were defined as showing causal associations with BPH at three-omics levels (FDR < 0.05 and PPH4 > 0.70); ii) Genes with evidence level 2 were defined as having causal associations with BPH at any two-omics levels (FDR < 0.05 and PPH4 > 0.70); iii) Genes with evidence level 3 were defined as having causal associations with BPH at any one-omics level (FDR < 0.05 and PPH4 > 0.70), and FDR < 0.05 in another one-omics level. Genes such as BTN3A2, NEU1, and C4A are located on chromosome 6, while LTBP3 is located on chromosome 11 (Fig. 1F). The findings revealed that BTN3A2 was classified as an evidence level 1 gene, C4A and NEU1 as evidence level 2 genes, and LTBP3 as an evidence level 3 gene (Fig. 1G). Notably, BTN3A2, as an evidence level 1 gene, demonstrated the strongest association across all examined levels. Furthermore, the genes at evidence level 2 and 3 were also found to be significant, with most of the evidence level genes focusing on methylation and gene expression levels (Fig. 1H).

Further, we performed the phenome-wide association studies (PheWAS) analysis, and the summarized data for each systemic disease in the analysis are presented in Table S8. The results indicated that BTN3A2 was associated with multiple diseases. BTN3A2 expression can influence the risk of diseases, such as Celiac disease, intestinal malabsorption (non-celiac), diffuse diseases of connective tissue, and thyrotoxicosis with or without goiter (Fig. 1I; Table S9). In the SMR analysis, BTN3A2 was identified as a risk factor for BPH. Therefore, if BTN3A2-targeted therapies are developed for BPH treatment in the future, patients may face an increased risk of developing skin and gastrointestinal disorders. Subsequently, we analyzed the expression of BTN3A2 in different tissues from the nTPM data of the Human Protein Atlas (HPA) cohort. The results showed that BTN3A2 was significantly expressed in the spleen, which was closely related to its regulation of the function of natural killer/T cells (Fig. 1J). At the same time, we further found through single-cell data clustering in the HPA database that BTN3A2 was highly expressed mainly in natural killer cells, T-cells, and plasma cells, and mainly served as a characteristic gene of natural killer cells (Fig. S1). Following this, we investigated the expression levels of BTN3A2 in BPH and adjacent tissues using data from the HPA database. As the Mendelian randomization analysis primarily draws from serum-based data, the tissue staining results were not as distinct. We appreciate your understanding of these limitations and remain committed to further exploring this area in future research (Fig. 1K). Additionally, evidence from blood-related diseases suggests that BTN3A2 protein levels are closely associated with susceptibility to infectious diseases (Fig. S2). Given that chronic prostatitis is commonly present in prostatic hyperplasia, this may represent a potential pathway for BTN3A2 to exert its effects.

BPH is the most prevalent urological condition among men over 50 years old, with chronic inflammation increasingly recognized as a key factor in its pathogenesis and progression. The etiology of chronic prostatitis remains poorly understood and may involve multiple concurrent stimuli.^3^ Recent studies indicate that histological inflammation correlates with International Prostate Symptom Score (IPSS) and National Institutes of Health-Chronic Prostatitis Symptom Index (NIH–CPSI) scores in BPH progression.^4^ In this study, we mainly used SMR and colocalization analyses to explore the causal role of IRGs in BPH, with the aim of identifying potential therapeutic targets. We confirmed the causal association between IRGs and BPH using SMR and colocalization analyses as the main tools and systematically explained the value of the immune-related genes in BPH. This study provides a new research target for future investigations and offers new insights and methodological approaches for BPH treatment through the level 1 evidence gene BTN3A2.

## CRediT authorship contribution statement

**Zhenpeng Zhu:** Writing – review & editing, Writing – original draft, Visualization, Data curation, Conceptualization. **Xing Ji:** Writing – original draft, Visualization, Methodology, Investigation. **Wenyuan Leng:** Visualization, Software, Formal analysis. **Weimin Hu:** Visualization, Software. **Chunru Xu:** Visualization, Methodology. **Xiaoyu Li:** Validation, Formal analysis. **Jinqin Qian:** Funding acquisition, Conceptualization. **Cuijian Zhang:** Writing – review & editing, Supervision, Conceptualization. **Jian Lin:** Writing – review & editing, Writing – original draft, Funding acquisition, Conceptualization.

## Data availability

All data generated or analyzed during this study are included in this published article and its additional files.

## Funding

This work was supported by grants from the 10.13039/501100001809National Natural Science Foundation of China (No. 82500819, 82270708) and Shenzhen Medical Research Fund (Guangdong, China) (No. A2302040).

## Conflict of interests

The authors declared no conflict of interests.
