# Supplementary material for: Multi-omics Mendelian randomization to identify novel immune therapeutic targets in benign prostatic hyperplasia
Source: Genes Dis. 2025 Nov 19;13(4):101944. doi: 10.1016/j.gendis.2025.101944 (PMC13015220; doi:10.1016/j.gendis.2025.101944)
Supplement: Multimedia component 1 [file mmc1.docx]

**Supplemental Material**

**Materials & methods**

**Study design and data collection**

The study design and data collection process are depicted in Figure 1. A set of immune-related genes was sourced from the innateDB (https://www.innatedb.com/) and Immuport (https://www.immport.org/shared/home) databases, comprising 2518 combined IRGs. Publicly available data for MR were acquired from the UK Biobank and FinnGen studies, and other extensive GWAS datasets. The UK Biobank cohort (ukb-b-11601) was employed as the discovery cohort, while the FinnGen R10 BPH cohort (Finngen_R10_N14_PROSTHYPERPLA) was utilized for replication and validation. Additionally, multi-omics data, including methylation, mRNA, and protein-related QTLs, were collected for subsequent analyses. The methylation quantitative trait loci (mQTL) of SNP-CpG data from blood samples were obtained from the McRae et al project, which included two cohorts BSGS (n = 614) and LBC (n = 1366) in peripheral blood , using Illumina HumanMethylation450 chips. The expression quantitative trait loci (eQTL) from blood samples were obtained from the eQTLGen Consortium, which encompasses 37 datasets and a total of 31,684 individuals. Concurrently, the protein quantitative trait loci (pQTL) related data from blood was obtained from the SomaScan v4 platform of the FinnGen R10 study, which contains 7156 proteins among 35,892 Iceland populations. In addition, eQTL data of BTN3A2 from blood and prostate tissue samples were procured from the GTEx Consortium.

**Exposure and outcome data preparing**

To select appropriate instrumental variables for analysis, we initially identified variables exhibiting a minor allele frequency (MAF) greater than 0.01 and a p-value less than 5×10^-8^. Consistent with prior studies, our selection of single-nucleotide polymorphisms (SNPs) was confined to those located within ± 1000kb gene region windows. Subsequently, the IRGs were selected from the QTLs data, which included 1464 methylated level genes with 7321 CpG sites, 1436 expression level genes, and 729 protein level genes. These were then subjected to further SMR and colocalization analyses. In conducting PheWAS analysis, the exposure data were obtained from the GTEx database and clumped using PLINK software with a cutoff of r^2^=0.1, kb=1000, and 503 European individuals' data from the 1000 Genomes Project were used as reference.

**SMR and Colocalization** **analyses**

The SMR and colocalization analyses were conducted in accordance with the protocol established by the Yang Lab. In comparison to the traditional MR analysis, the SMR analysis is associated with a greater statistical power. In this study, we utilized SMR analyses to explore the casual association between methylation levels, expression levels, protein levels of IRGs, and the risk of BPH. The top 20 SNPs in the cis-QTL region with a P-value threshold of 5.0×10^−8^ and a corresponding gene windows threshold of ±500kb (methylation levels), ±1000kb (expression levels), and ±1000kb (protein levels) were screened for further HEIDI tests. Allele frequencies that differed by more than 0.2 and had a maximum proportion exceeding 0.05 were excluded. Then, the SNPs with p-value of SMR < 0.05 and p-HEIDI > 0.01 were selected for the colocalization analyses.

Colocalization analysis was performed using the Coloc R package to link summary stats from two distinct datasets of unrelated individuals, utilizing SNP identifiers. Five mutually exclusive hypotheses regarding association and genetic sharing in the region were proposed: (1) H0: No association to both traits; (2) H1: Association to trait 1 only; (3) H2: Association to trait 2 only; (4) H3: Association to both traits, distinct causal variants; (5) H4: Association to both traits, shared causal variants; The prior probabilities of p1, p2, p12 were set as 10^-4^, 10^-4^, 10^-5^ ,respectively. Meanwhile, the colocalization with posterior probability of H4 (PPH4) > 0.70 and false discovery rate (FDR) < 0.05 were considered strong colocalization.

**Pathway enrichment analysis of significant IRGs**

In the present study, we selected IRGs with FDR less than 0.05 in SMR analysis as genes significantly associated with BPH. We first utilized the GeneMania database (https://genemania.org/) to construct a functional network of target genes and search for pathways associated with these target genes in its database. We then performed GO, KEGG, and DO pathway enrichment analyses among these genes using the clusterProfiler and org.Hs.eg.db R packages and plotted the data using the XianTao tools (<https://www.xiantaozi.com/>).

**Multi-omics level analysis of the evidence level genes**

To clarify the potential regulatory role of IRGs in the entire central dogma process on BPH, we explored three types of gene regulation evidence based on previous study. We divided causal effects of IRGs on BPH into three evidence levels based on the results obtained in mQTL, eQTL, and pQTL: (1) Genes with evidence level 1 are defined as having causal associations with BPH at three-omics levels (FDR < 0.05 and PPH4 > 0.70); (2) Genes with evidence level 2 are defined as having causal associations with BPH at any two-omics levels (FDR < 0.05 and PPH4 > 0.70) (3) Genes with evidence level 3 are defined as having causal associations with BPH at any one-omics level (FDR < 0.05 and PPH4 > 0.70), and FDR<0.05 in another one-omics level.

**PostGWAS analyses of BTN3A2**

In this study, we further explored the causal effects of BTN3A2 gene as specific exposure factors in a range of outcomes, including Lee_UKBB data, according to the AstraZeneca PheWAS Portal (https://azphewas.com/) and the PheWeb database (https://pheweb.org/). Further clarification is needed to determine whether BTN3A2 has significant relevance in common diseases apart from BPH. Further, we performed a further exploration of BTN3A2 using the Human Protein Atlas (HPA) database (<https://www.proteinatlas.org/>). Through the HPA database, we have determined the distribution and expression of BTN3A2 in different tissues. In the HPA database, we obtained the cluster of BTN3A2 as a characteristic gene expression through single-cell mapping. At the same time, we obtained the expression of BTN3A2 in blood among different diseases, as well as the protein status of BTN3A2 in prostate tissue obtained through the HPA database.

**Figure S1-S2**

**
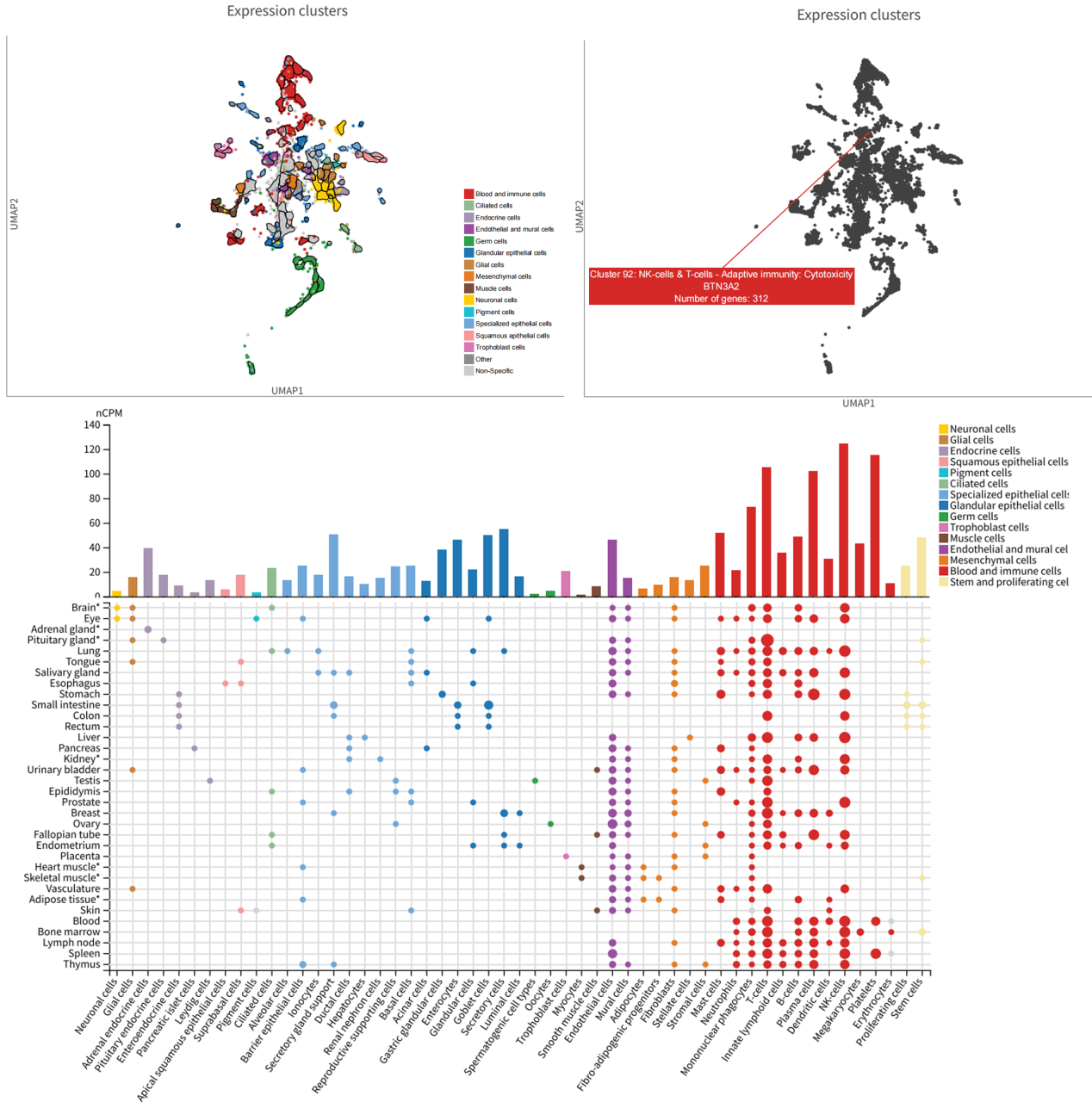
**

**Figure S1.** Distribution of BTN3A2 in tissues from the HPA database. (A) The colors indicate different cell clusters. Filter to select the BTN3A2 signature gene cluster. (B) The expression level of BTN3A2 in different cells of different tissues is expressed as nCPM. The size of the circles indicates the level of expression, and the color indicates the different cell types. HPA, Human Protein Atlas; CPM, counts per million.


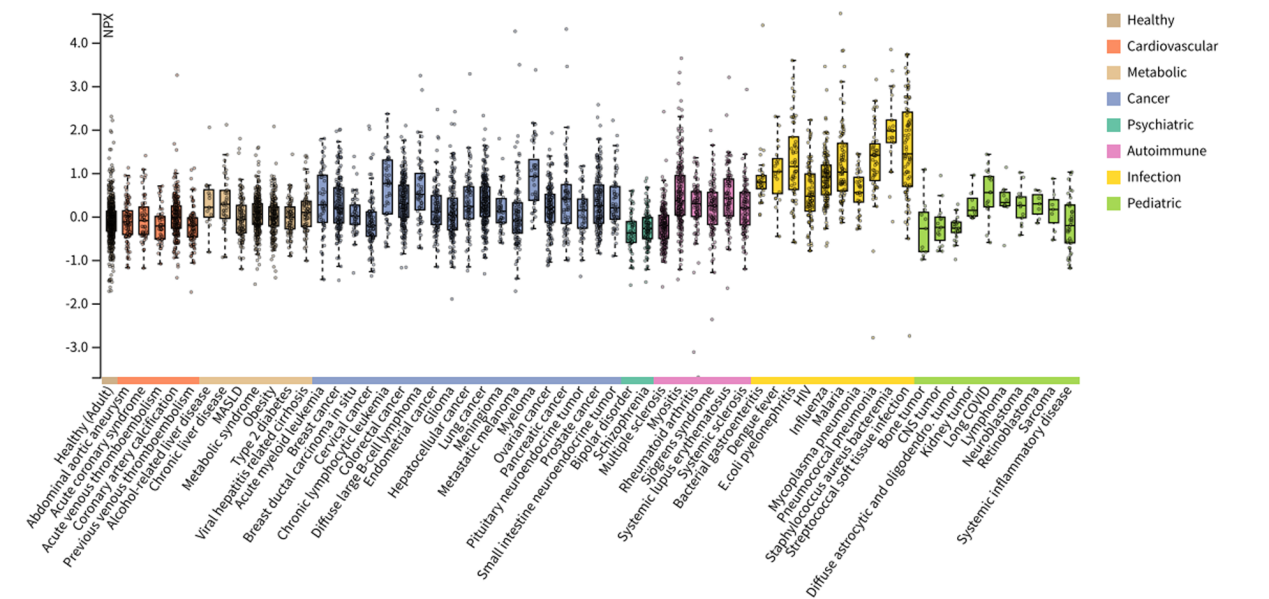


**Figure S2.** Changes in BTN3A2 protein expression levels in the blood for different types of disease. Different types of diseases are marked with different colors.
